# Support for Thrombolytic Therapy for Acute Stroke Patients on Direct Oral Anticoagulants: Mortality and Bleeding Complications

**DOI:** 10.5811/westjem.18063

**Published:** 2024-04-08

**Authors:** Paul Koscumb, Luke Murphy, Matthew Talbott, Shiva Nuti, George Golovko, Hashem Shaltoni, Dietrich Jehle

**Affiliations:** * University of Texas Medical Branch, Department of Emergency Medicine, Galveston, Texas; † University of Texas Medical Branch, Department of Pharmacology and Toxicology, Galveston, Texas; ‡ University of Texas Medical Branch, Department of Neurology, Galveston, Texas

## Abstract

**Background:**

Alteplase (tPA) is the initial treatment for acute ischemic stroke. Current tPA guidelines exclude patients who took direct oral anticoagulants (DOAC) within the prior 48 hours. In this propensity-matched retrospective study we compared acute ischemic stroke patients treated with tPA who had received DOACs within 48 hours of thrombolysis to those not previously treated with DOACs, regarding three outcomes: mortality; intracranial hemorrhage (ICH); and need for acute blood transfusions (as a marker of significant blood loss).

**Methods:**

Using the United States cohort of 54 healthcare organizations in the TriNetx database, we identified 8,582 stroke patients treated with tPA on DOACs within 48 hours of thrombolysis and 46,703 stroke patients treated with tPA not on DOACs since January 1, 2012. We performed propensity score matching on demographic information and seven prior clinical diagnostic groups, resulting in a total of 17,164 acute stroke patients evenly matched between groups. We recorded mortality rates, frequency of ICH, and need for blood transfusions for each group over the ensuing 7- and 30-day periods.

**Results:**

Patients treated with tPA on DOACs had reduced mortality (3.3% vs 7.3%; risk ratio [RR] 0.456; *P* < 0.001), fewer ICHs (6.8% vs 10.1%; RR 0.678; *P* < 0.001), and less risk of major bleeding as measured by frequency of blood transfusions (0.5% vs 1.5%; RR 0.317; *p* < 0.001) at 7 days post thrombolytic, than the tPA patients not on DOACS. Findings for 30 days post-thrombolytics were similar/statistically significant with lower mortality rate (7.2% vs 13.1%; RR 0.550; *P* < 0.001), fewer ICHs (7.6% vs 10.8%; RR 0.705; *P* < 0.001), and fewer blood transfusions (0.9% vs 2.0%; RR 0.448; *P* < 0.001).

**Conclusion:**

Acute ischemic stroke patients treated with tPA who received DOACs within 48 hours of thrombolysis had lower mortality rates, reduced incidence of ICH, and less blood loss than those not on DOACs. Our study suggests that prior use of DOACs should not be a contraindication to thrombolysis for ischemic stroke.

Population Health Research Capsule What do we already know about this issue?

*While tPA is the initial treatment for ischemic stroke, guidelines exclude patients taking direct oral anticoagulants (DOAC) due to theoretical risk of bleeding.*

What was the research question?

*Do patients taking DOACs who receive tPA have worse outcomes than those not taking anticoagulants?*

What was the major finding of the study?

*With DOAC there was reduced mortality (3.3% vs 7.3%; RR 0.456; P < 0.001) and intracerebral hemorrhage (6.8% vs 10.1%; RR 0.678; P < 0.001)*

How does this improve population health?

*Use of DOACs is increasing; this should not prevent patients taking DOACs from receiving the benefits of reperfusion therapy for ischemic stroke.*

## INTRODUCTION

In the United States, stroke remains common, with the estimated risk of stroke over an individual’s lifetime approaching one in four. Ischemic stroke represents 87% of acute insults with intracerebral hemorrhage and subarachnoid hemorrhage making up the remaining balance.^1^^,^^2^ For patients who are eligible, the mainstay of treatment of ischemic stroke is restoration of blood flow via thrombolytics and/or thrombectomy. Alteplase (tPA) is currently the only thrombolytic approved for use in acute ischemic stroke by the US Food and Drug Administration.^3^

Thromboembolism from atrial fibrillation is a frequent cause of ischemic stroke and becomes particularly prevalent with aging. In the Framingham study, atrial fibrillation represented a 1.5% risk of stroke in the 50–59 age group and rose to a 23.5% risk in the 80–89 age group.^4^ As a preventative measure, most patients with atrial fibrillation are treated with anticoagulation, which can effectively reduce the risk of stroke by approximately two-thirds when compared to placebo.^5^^–^^9^ Patients with valvular atrial fibrillation should be treated with oral vitamin K antagonists (VKA).^10^^,^^11^ However, direct oral anticoagulants (DOAC), which include direct thrombin inhibitors and factor Xa inhibitors, have demonstrated non-inferiority to VKAs in the prevention of acute ischemic stroke in patients with non-valvular atrial fibrillation.^12^^–^^15^

The most recent 2019 update to the 2018 American Heart Association (AHA) guidelines on tPA excludes patients with concomitant usage of DOACs within 48 hours of the last dose intake, unless coagulation parameters are obtained and normal. Coagulation parameters include tests such as activated partial thromboplastin time (aPTT), prothrombin time (PT), platelet count, thrombin time (TT), or direct factor Xa activity assay.^16^ Recent data has suggested that the use of DOACs may not increase the risk of symptomatic intracerebral hemorrhage even in the absence of reversal agents,^17^^–^^20^ but this has not yet led to a change in guidelines. Prompt administration of thrombolytics to patients with acute ischemic stroke has the potential to lead to clot breakdown and, ideally, restored cerebral perfusion. Patients with acute ischemic stroke who are treated with thrombolytics have improved neurological outcome at three months^21^^,^^22^ and have a lower risk of long-term mortality^23^; therefore, it is critical to identify the maximum number of patients that can safely receive this intervention. The use of DOACs has rapidly increased in the past decade both for primary stroke prevention in patients with atrial fibrillation, and in the treatment of venous thromboembolism.^24^^,^^25^ Therefore, this represents a large cohort of patients who may be deprived of the potential benefits of reperfusion therapy.

We sought to assess whether patients receiving DOACs who also received tPA would have an increased risk of adverse events. We evaluated mortality and rate of hemorrhagic conversion across a large, retrospective dataset. Due to the nature of the healthcare dataset, we were not able to assess for severity of all other bleeding directly. Thus, we evaluated for whether patients required blood transfusion as a surrogate marker for clinically significant bleeding. We analyzed these three outcomes at 7 and 30 days post thrombolytic.

## METHODS

TriNetX is a global federated health research network providing de-identified access to retrospective electronic health records (diagnoses, procedures, medications, lab values, genomic information) from approximately 91 million patients in 54 large healthcare organizations (HCO) within the United States.^26^ In this study we used the US Collaborative Network to identify patients who were treated with tPA for acute stroke. Two cohorts were identified for this study within the group of patients treated with tPA on the day of or within one day of the diagnosis of acute stroke: Cohort 1 consisted of acute stroke patients treated with tPA who had received a DOAC on the day of or one day prior to their presentation with acute stroke; and Cohort 2 consisted of actue stroke patients treated with tPA who had not received a DOAC within seven days of the diagnosis of stroke.

We identified stroke patients of all ethnicities, races, and genders using International Classification of Diseases, 10^th^ modification (ICD-10) code I63 (Cerebral Infarction, 1.49 million cases). In the database there were 565,835 patients who were treated with tPA using RxNORM:8410. The dataset was limited to those patients whose index event occurred on or after January 1, 2012, and who had the diagnosis of cerebral infarction so as to exclude patients treated with tPA for acute coronary syndrome, pulmonary embolism, etc. We then generated two cohorts. In Cohort 1, patients had received a DOAC (edoxaban, dabigatran, apixaban, or rivaroxaban) on the day of or one day prior to their stroke and thrombolytic using RxNORM 1599538, 1037042, 1364430, or 1114195, resulting in 8,582 patients. Cohort 2 was defined as stroke patients treated with tPA who were documented to not be on DOACs in the prior 7 days, resulting in 46,703 patients.

To control for potentially confounding risk factors for the measured outcomes, we performed propensity score matching based on the age at stroke diagnosis, race, ethnicity, gender, presence of hypertensive diseases (ICD-10 codes I10-I16), diabetes mellitus (E08-E13), acute kidney failure and chronic kidney disease (N17-N19), overweight status/obesity (E66), heart failure (I50), cardiac arrest (I46), and ischemic heart diseases (I20-I25). We used the balanced cohort tool in TriNetX for matching.^26^

We performed the outcome analysis between the two cohorts for three events: death (vital status: deceased); nontraumatic intracranial hemorrhage (ICH) and blood transfusions (Current Procedural Terminology code 36430). Nontraumatic ICH was defined as nontraumatic subarachnoid – ICD-10 code I60; nontraumatic intracerebral hemorrhage – ICD-10:I61, or nontraumatic acute subdural hemorrhage – ICD-10:I62.01. Rates of blood transfusion were used as a marker of significant blood loss post thrombolytic administration. All tested outcomes occurred on or after the day of diagnosis of stroke. We measured outcomes over a period of 7 and 30 days post thrombolytics. Patients who had the outcome at the time of or prior to the designated time window were subsequently excluded from the analysis.^26^

We performed univariate analysis using the measure-of-association tool in TriNetX, which compares outcomes within the designated time frames for each cohort reported both as risk ratios (RR), odds ratios, 95% confidence intervals (CI) of these ratios and risk difference as a *P*-value. We obtained de-identified patient data from the TriNetX US Collaborative Network database on November 4, 2022, and we performed the data analyses on the same date. We reported our outcomes as RRs with 95% CIs and risk differences. The TriNetX platform provides access to aggregated counts and statistical summaries of de-identified patient records. No protected health information or personal data is available to the platform users; therefore, this project was exempt from institutional review board review (www.trinetx.com).^26^ Our manuscript follows STROBE guidelines for observational cohort studies.^27^

## RESULTS

We identified 91,1707,410 patients in the TriNetX United States Collaborative Network from 54 academic medical centers/healthcare organizations. In Cohort 1 of patients treated with tPA on DOACs within 48 hours of thrombolysis for stroke we identified 8,582 patients. In Cohort 2 of patients treated with tPA for stroke documented to not be on DOACs in the prior seven days, there were 46,703 patients. After propensity score matching on basic demographic information and seven prior clinical diagnostic groups associated with mortality there were a total of 17,164 acute stroke patients evenly matched between the DOAC and no-DOAC groups.

Most of the demographic groups except for gender (male/female) were statistically different between the two cohorts before matching. All pre-existing medical conditions associated with mortality were statistically different between cohorts. After propensity matching most of the demographic groups or pre-existing medical conditions were statistically different between the cohorts. Tables 1 and 2 present the demographic characteristics and pre-existing conditions in Cohorts 1 and 2 before and after propensity matching. TriNetX reports infrequent events with outcomes that are ≤10 as 10; so the difference between the two cohorts may have been slightly greater for the Native American and Hawaiian demographic groups where the number in the DOAC group is listed as 10.

**Table 1.** Cohort 1 (N = 8,582) and Cohort 2 (N = 46,703) characteristics before propensity score matching.

Demographics						
Cohort		Mean ± SD	Patients	% of cohort	*P*-value	Std diff.
1 2	Age at Index	68.7 +/− 14.4 64.1 +/− 16.7	8,582 46,703	100% 100%	<0.001	0.30
1 2	Male		4,382 23,910	51.1% 51.2%	0.82	0.003
1 2	Female		4,199 22,790	48.9% 48.8%	0.82	0.003
1 2	Not Hispanic or Latino		7,457 34,166	86.9% 73.2%	<0.001	0.35
1 2	White		6,551 32,325	76.3% 69.2%	<0.001	0.16
1 2	Unknown Ethnicity		875 10,576	10.2% 22.6%	<0.001	0.34
1 2	Black or African American		1,32 38,643	15.4% 18.5%	<0.001	0.08
1 2	Unknown Race		557 4,731	6.5% 10.1%	<0.001	0.13
1 2	Hispanic or Latino		250 1,961	2.9% 4.2%	<0.001	0.07
1 2	Asian		117 690	1.4% 1.5%	0.42	0.01
1 2	American Indian or Alaska Native		25 230	0.3% 0.5%	0.01	0.03
1 2	Native Hawaiian or Other Pacific Islander		10 84	0.1% 0.2%	0.19	0.02

Diagnosis							
Cohort	ICD-10	Pre-existing condition	Mean ± SD	Patients	% of cohort	*P*-value	Std diff.
1 2	I10-I16	Hypertensive diseases		7,001 24,784	81.6% 53.1%	<0.001	0.64
1 2	I20-I25	Ischemic heart diseases		4,227 12,330	49.3% 26.4%	<0.001	0.49
1 2	E08-E13	Diabetes mellitus		3,638 12,864	42.4% 27.5%	<0.001	0.32
1 2	N17-N19	Acute kidney failure and chronic kidney disease		3,422 10,877	39.9% 23.3%	<0.001	0.36
1 2	E66	Overweight and obesity		2,728 8,343	31.8% 17.9%	<0.001	0.33
1 2	I50	Heart failure		3,225 7,805	37.6% 16.7%	<0.001	0.48
1 2	I46	Cardiac arrest		297 963	3.5% 2.1%	<0.001	0.09

**Table 2.** Cohort 1 (N = 8,582) and Cohort 2 (N = 8,582) characteristics after propensity score matching.

Demographics						
Cohort		Mean ± SD	Patients	% of cohort	*P*-value	Std diff.
1 2	Age at Index	68.7 +/− 14.4 68.8 +/− 14.3	8,582 8,582	100% 100%	0.78	0.004
1 2	Male		4,382 4,351	51.1% 50.7%	0.64	0.007
1 2	Female		4,199 4,231	48.9% 49.3%	0.63	0.007
1 2	Not Hispanic or Latino		7,457 7,557	86.9% 88.1%	0.02	0.04
1 2	White		6,551 6,677	76.3% 77.8%	0.02	0.04
1 2	Black or African American		1,323 1,301	15.4% 15.2%	0.64	0.007
1 2	Unknown Ethnicity		875 808	10.2% 9.4%	0.09	0.03
1 2	Unknown Race		557 484	6.5% 5.6%	0.02	0.04
1 2	Hispanic or Latino		250 217	2.9% 2.5%	0.12	0.02
1 2	Asian		117 101	1.4% 1.2%	0.28	0.02
1 2	American Indian or Alaska Native		25 16	0.3% 0.2%	0.16	0.02
1 2	Native Hawaiian or Other Pacific Islander		10 10	0.1% 0.1%	>0.99	<0.001

Diagnosis							
Cohort	ICD-10	Pre-existing condition	Mean ± SD	Patients	% of cohort	*P*-value	Std diff.
1 2	I10-I16	Hypertensive diseases		7,001 7,048	81.6% 82.1%	0.35	0.01
1 2	I20-I25	Ischemic heart diseases		4,227 4,206	49.3% 49.0%	0.75	0.01
1 2	E08-E13	Diabetes mellitus		3,638 3,663	42.4% 42.7%	0.70	0.006
1 2	N17-N19	Acute kidney failure and chronic kidney disease		3,422 3,374	39.9% 39.3%	0.45	0.01
1 2	I50	Heart failure		3,225 3,047	37.6% 35.5%	0.01	0.04
1 2	E66	Overweight and obesity		2,728 2,642	31.8% 30.8%	0.16	0.02
1 2	I46	Cardiac arrest		297 248	3.5% 2.9%	0.03	0.03

We excluded patients if they had an outcome at the time of or prior to the designated index event based on what is recorded in the health records. The risk analysis for the mortality outcome had 193 patients excluded from Cohort 1 (DOAC) and 171 patients from Cohort 2 (no-DOAC). These exclusions are necessary when the timing of an outcome diagnosis is uncertain or occurs before the time window. These exclusions are also in part necessary when the outcome and index event occurs within hours of each other, as the TriNetX database does not always have the degree of granularity to distinguish which event occurred first. The DOACs were much less frequently used more than 10 years ago (introduced approximately 13 years ago); thus, we eliminated this period from the treatment and control groups to avoid confounding.

Patients treated with tPA on DOACs were found to have reduced mortality (3.3% vs 7.3%; RR 0.456; *P* < 0.001), lower incidence of ICH (6.8% vs 10.1%; RR 0.678; *P* < 0.001), and less risk of major bleeding as measured by frequency of blood transfusions (0.5% vs 1.5%; RR 0.317; *P* < 0.001) at seven days post thrombolytic, than the tPA patients who had not been on DOACS. We found similar statistically significant findings with lower mortality rate (7.2% vs 13.1%; RR 0.550; *P* < 0.001), lower incidence of ICH (7.6% vs 10.8%; RR 0.705; *P* < 0.001), and fewer blood transfusions (0.9% vs 2.0%; RR 0.448; *P* < 0.001) at 30 days after the administration of the thrombolytic agent in the 10-year dataset. This information regarding the patient outcomes at seven days (Table 3) and 30 days (Table 4) post thrombolytic is below. The 95% CIs for the RR of death, ICH, and significant other bleeding are also presented in these tables.

**Table 3.** Outcomes at 7 Days after propensity score matching.

Outcome	tPA + DOAC^*^ (n = 8,582)	tPA - DOAC (n = 8,582)	Risk Ratio (95% CI^‡^)	Probability (p)
Mortality	3.3%	7.3%	0.456 (0.398, 0.524)	<0.001
ICH^†^	6.8%	10.1%	0.678 (0.609, 0.756)	<0.001
Blood Transfusion	0.5%	1.5%	0.317 (0.220, 0.456)	<0.001

^*^ Direct Oral Anticoagulant.

^‡^ Confidence Interval.

^†^ Intracranial Hemorrhage.

**Table 4.** Outcomes at 30 Days after propensity score matching.

Outcome	tPA + DOAC^*^ (n = 8,582)	tPA - DOAC (n = 8,582)	Risk Ratio (95% CI^‡^)	Probability (p)
Mortality	7.2%	13.1%	0.550 (.500, 0.604)	<0.001
ICH^†^	7.6%	10.8%	0.705 (0.636, 0.781)	<0.001
Blood Transfusion	0.9%	2.0%	0.448 (0.341, 0.590)	<0.001

## DISCUSSION

In this large, multicenter, propensity-matched, retrospective study, patients with ischemic stroke who received tPA and had received DOACs within two days of thrombolytics were found to have significantly lower risk of death, ICH, and bleeding when compared to patients who received tPA without prior DOACs. These findings were statistically significant at both 7 and 30 days post-thrombolytic. This is significant because current stroke guidelines recommend against administration of thrombolysis in patients who have taken a DOAC within 48 hours of presentation due to a theoretical increased risk of bleeding. Although prior studies have indicated that DOAC use may not increase the risk of intracerebral hemorrhage, to our knowledge our study is the first to suggest a decreased risk of bleeding as well as a decreased risk of death.

Recommendations for withholding thrombolytics from patients using DOACs in the absence of lab testing such as activated partial thromboplastin time (aPTT), prothrombin time (PT), platelet count, thrombin time (TT_, or direct factor Xa activity assay were first instituted in 2013 based on consensus opinion with limited-to-no data (Class IIIC recommendation).^28^ These recommendations may have been prudent at the time as DOACs were a relatively novel class of medication, and data surrounding their use was only beginning to emerge. However, the clinical utility of many common coagulation parameters in patients on DOACs is limited. The APTT, PT, and TT are readily available but poorly sensitive and specific for monitoring of DOACs and should not be used quantitatively to evaluate the degree of anticoagulation effect. By contrast, plasma drug concentration and anti-factor Xa assays may quantify the degree of anticoagulation but are not always available and may require specialized laboratory send-out testing.^29^ At our institution, dilute TT is only performed twice weekly, and anti-factor Xa for DOACs is not performed by our hematology lab despite our institution being a comprehensive stroke center. We suspect that this is similar to many comprehensive stroke centers nationwide. Awaiting the above results is problematic since tPA administration is time sensitive; therefore, patients are functionally excluded from receiving tPA due to the time required to obtain these studies.

It has been known for nearly a decade now that DOAC use is non-inferior to warfarin in the prevention of acute ischemic stroke in patients with nonvalvular atrial fibrillation.^12^^–^^15^ However, while adequate anticoagulation decreases the risk of stroke, it does not completely negate the risk, with the estimated annual risk of ischemic stroke despite oral anticoagulation being approximately 1–2%.^30^ We conjecture that perhaps this cohort of patients, while still experiencing ischemic stroke, has relatively milder strokes with smaller thrombus burden, which puts these patients at lower risk of death regardless of tPA administration. Furthermore, these patients may have smaller areas of infarction, which may put them at a diminished risk of hemorrhagic conversion due to less volume of fragile tissue.

Our results favoring DOACs are in line with other data that also suggest that DOACs are associated with lower risk of fatal or disabling stroke when compared to coumadin.^31^ Further study in this area could seek to stratify patients by initial National Institutes of Health Stroke Scale score or volume of infarction on imaging to confirm these hypotheses. These hypotheses do not fully explain the third outcome demonstrating a lower rate of blood transfusion as a surrogate for extracranial bleeding. It is possible that less debilitating strokes among patients on DOACs could place less metabolic stress on the body and result in a less globally critical condition of the patient. It is known, for example, that length of intensive care unit stay increases the risk of gastrointestinal bleeding.^32^ If patients receiving DOACs have smaller strokes and less severe disease resulting in decreased multisystem organ failure, then this may account for the differences seen in our dataset. It is also possible that the limited half-life of DOAC medications allows for a reduction in their bleeding effect even with a time of abstinence less than 48 hours. The maximal half-life of rivaroxaban is approximately 12 hours, and that of apixaban is similar. Warfarin’s mean half-life on the other hand is 40 hours, and this can vary widely.

The rate of ICH seen in this study is slightly higher than was seen in prior studies, for example, in the NINDS trial where the symptomatic ICH rate was found to be 6.4% in patients treated with thrombolytics. This was defined as “any CT [computed tomography]-documented hemorrhage that was temporally related to deterioration in the patient’s clinical condition in the judgment of the clinical investigator” within 36 hours of treatment.^21^ Our study evaluated for outcomes at 7 and 30 days following treatment, and the more expansive time frame may partially explain the increased hemorrhage rate seen in our study. Additionally, our study included all ICH, not only symptomatic ICH. Institutional stroke protocols may mandate for a routine CT brain for all patients treated with thrombolytics regardless of whether they were symptomatic, and this may have captured patients included in our study who would have been excluded from others.

## LIMITATIONS

The retrospective cohort design of this study makes establishing causality difficult. However, to our knowledge our study is much larger than any other in the literature evaluating outcomes after tPA in patients taking DOACs vs those not taking DOACs. The size of our study, combined with the propensity matching that we performed on our sample, gives it more power to evaluate for differences in outcomes. Furthermore, the generalizability of our study is increased by the number of institutions queried by TriNetX.

We performed 1:1 propensity matching for age, gender, race, hypertensive diseases, ischemic heart disease, diabetes mellitus, acute and chronic kidney failure, heart failure, overweight status/obesity, and prior cardiac arrest, as these are known risk factors for mortality. It is important to note that although we selected multiple potentially confounding variables for matching, a variable that we did not include could have confounded the relationship between drug treatment and mortality. Authors that have been critical of propensity matching techniques acknowledge that most potential deficiencies of this technique are minimized in larger datasets such as this one.

Additionally, we used blood transfusion as a surrogate marker of significant hemorrhage, although this cannot be determined to be secondary to the thrombotic administration. This outcome was investigated over a period of 7 and 30 days post-thrombotic treatment, which should limit confounding due to other causes of bleeding.

We did not exclude warfarin use from our two patient populations. It is possible, but unlikely, that the presence of warfarin use in some non-DOAC patient cohort may have skewed toward higher rates of ICH. However, 2019 AHA guidelines specifically recommend against treatment of ischemic strokes with thrombolytics in patients with an international normalized ratio >1.7, which would exclude most patients therapeutic on warfarin. In addition, patients taking warfarin are unlikely to be concurrently treated with a DOAC.

We are unsure why the patients included in this study were treated with thrombolytics, contrary to current stroke guidelines. It is possible that these were patients who, through coagulation assays such as direct factor Xa, were found not to be anticoagulated. However, due to the time-consuming nature of these studies and the need to administer thrombolytics in a time-sensitive manner and their limited availability even at comprehensive stroke centers such as our own, we believe it is unlikely that these patients were included in a systemic manner or in large numbers. We were unable to evaluate whether there was a clustering of patients on DOACs who received thrombolytics at certain centers, as the TriNetX privacy policy does not allow us to view this information due to the de-identified nature of the dataset. The de-identified nature of the dataset also required us to be dependent on accurate input of the electronic health record to evaluate whether or not the patient was taking a DOAC.

## CONCLUSION

In this large, retrospective, multicenter study, patients taking DOACs who received tPA for acute ischemic stroke had a reduced risk of death, lower incidence of ICH, and decreased blood loss in comparison to those who received tPA and were not taking DOACs. Our study adds to the increasing evidence that DOAC use should not be a contraindication to thrombolytics in the initial treatment of acute ischemic stroke. The stroke guidelines should be updated to reflect these findings.
